# Preparation and Characterization of Amphoteric Cellulose Hydrogels as Adsorbents for the Anionic Dyes in Aqueous Solutions

**DOI:** 10.3390/gels1010094

**Published:** 2015-08-24

**Authors:** Hiroyuki Kono

**Affiliations:** Department of Science and Engineering for Materials, Tomakomai National College of Technology, Tomakomai, Hokkaido 059 1275, Japan; E-Mail: kono@sem.tomakomai-ct.ac.jp; Tel./Fax: +81-144-67-8036

**Keywords:** amphoteric hydorogels, cellulose ampholyte, adsorption of dyes, kinetic analysis, adsorption isotherm

## Abstract

A series of cellulose ampholytes (CAms), with substitution degrees of cationic groups (DS_C_) of 0.41, 0.79, and 1.08, and with a substitution degree of anionic groups of 0.68, was prepared from sodium carboxymethylcellulose by a cationization reaction with 2,3-epoxypropyltrimethylammonium chloride. The obtained CAms were crosslinked by ethyleneglycoldiglycidylether (EGDE) to obtain cellulose polyampholyte hydrogels (CAmGs). The CAmGs adsorbed three anionic dyes, *viz.* AR9, AR13, and AB92, and the absorption depended on the DS_C_ of the hydrogels and the pH of the adsorption medium: the maximum adsorption of anionic dyes occurred using CAmGs with higher DS_C_ values, and under lower pH (less than 3) conditions. The adsorption of these dyes can be fitted by the Langmuir adsorption isotherm model, which revealed the maximum flocculation capacity of CAmGs for each dye. These encouraging results indicate that CAmGs may be applicable for use as novel adsorbents for wastewater treatment.

## 1. Introduction

More than about 7 × 10^5^ tons of dyes are annually used in the chemical industry worldwide, about 10%–15% of which are discharged as effluents that seriously pollute the environment and affect humans as well as aquatic organisms [1,2]. There are more than 10 thousand types of commercially available dyes, most of which are considered to be toxic [2,3,4]. Most of the dyes have complex aromatic structures that are very stable, and thus accumulate in nature [5,6,7]. Therefore, the removal of dyes from aqueous solutions is environmentally important. Among several common chemical and physical methods, the adsorption process is one of the effective techniques that has been successfully employed for color removal from wastewater [8,9]. Many adsorbents have been tested for their potential to lower dye concentrations in aqueous solutions [1,10,11,12]. Activated carbon, in particular, is conventionally used to adsorb dyes from wastewater. However, regeneration of the activated carbon is difficult, despite its relatively low cost, which restricts its use [13].

Recently, much attention has been paid to dye removal from water via adsorption using natural polysaccharide-based hydrogels prepared from sodium alginate [14], carrageenan [15], pectin [16], and others because of their biocompatibility and biodegradability. Because of the anionic properties of these polysaccharides, these hydrogels can adsorb cationic dyes from aqueous solutions with the electrostatic attraction force. For the adsorption of anionic dyes, chitosan-based hydrogels have been studied because the amino groups of chitosan are positively charged in acidic solutions due to the protonation of these groups. However, there are very few cationic hydrogels for the adsorption of anionic dyes because there are no naturally occurring cationic polysaccharides other than chitosan [17].

From the viewpoint of the quantity of various polysaccharides occurring in nature, cellulose, which is the most abundant natural polymer on earth, has great potential as one of the most environmentally friendly non-food sources for the production of a wide range of eco-friendly products. The number of potential chemical modifications of cellulose also makes it an attractive adsorbent hydrogel candidate. For example, sodium carboxymethyl cellulose (CMC), which is well known as a water-soluble cellulose derivative and exhibits characteristic anionic properties, has shown promising results as an anionic flocculation agent [18]. The hydrogels prepared from CMC with a bifunctionalized crosslinking agent exhibit adsorption of cationic dyes [19]. In addition, a quaternary ammonium sodium salt derivative of cellulose was prepared by the homogeneous reaction of cellulose with 2,3-epoxypropyl trimethyl ammonium chloride (EPTMAC) as a cationization reagent. The cationic cellulose was shown to be an excellent flocculant for a kaolin suspension. Therefore, cationic cellulose is expected to be one of the most effective candidates for forming the base matrix of the hydrogels for cationic dye adsorption [20].

In general, cellulose has a highly crystallized structure due to inter- and intramolecular hydrogen bonds between its hydroxyl groups. For the preparation of cellulose derivatives in homogeneous reaction systems, therefore, solvents such as an aqueous NaOH/urea solution, LiCl/DMSO, tetra-*n*-butylammonium fluoride/DMSO, or specific ionic liquids are required [21]. The dissolution process for cellulose with most of these solvents is time-consuming and/or requires preswelling with a sequential solvent change and heating. In the case of a heterogeneous reaction system, the reaction efficiency of cellulose is considerably lower than that in a homogeneous reaction system because the reaction proceeds from the surface of the cellulose fibrils. In one of the methods for the preparation of cellulose derivatives in a homogeneous reaction system, the cellulose derivatives, which could be dissolved or swollen in versatile solvents, are used as the starting material. For example, a series of cellulose ampholytes (CAms), for which hydroxyl groups had been substituted for cationic and anionic groups, were homogeneously prepared from CMC with an anionic carboxymethyl substitution degree (DS_A_) of 0.6 in aqueous alkaline solution [22]. The DS_C_s of the obtained CAms were in the range of 0.24–1.02, and their DS_A_s were all 0.60. The CAms behaved like cationic polymers in acidic solution because of the protonation of their carboxymethyl groups, which permits amphoteric cellulose to be a cationic flocculant for negatively charged molecules. Also, the CAms for which the DS_C_ was more than the DS_A_ could flocculate negatively charged molecules in neutral and alkaline solutions as well.

**Figure 1:**
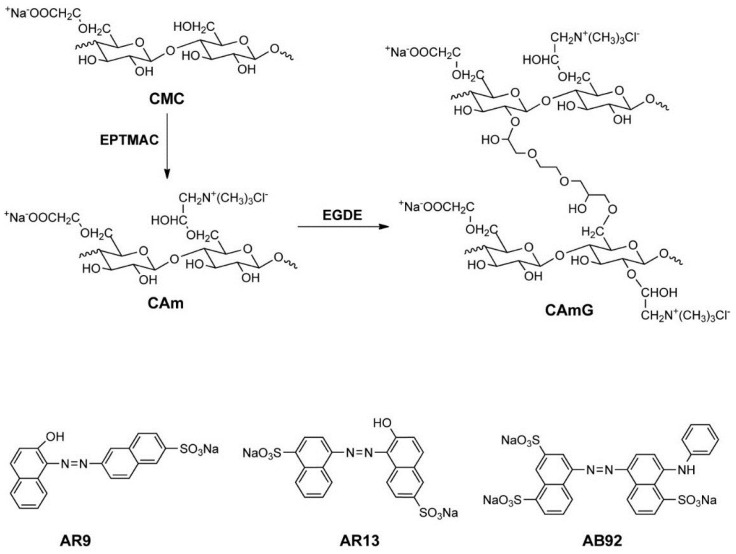
Scheme for CAm and CAmG synthesis from CMC (**top**) and structures of anionic dyes used in this study (**bottom**). In this scheme, although cationization at the CMC C6 hydroxyl group is illustrated, cationization also can occur at other unsubstituted hydroxyl groups of CMC. In addition, crosslinking can potentially occur in any hydroxyl groups of CAm. These possibilities were omitted in the scheme for simplicity.

In this study, as shown in Figure 1, novel amphoteric hydrogels were prepared from a series of CAms having different DS_C_ values, and the adsorption ability of the prepared CAmGs for three anionic dyes, *viz.* AR9, AR13, and AB92, was investigated in order to reveal their potential as effective adsorbents for anionic pollutants. The effects of the cationic substitution degree and the pH on the adsorption of the anionic dyes onto the CAmGs, and the kinetics and isotherms of the adsorptions, were also investigated and compared in detail for the elucidation of the adsorption mechanism, which is described herein.

## 2. Results and Discussion

### 2.1. Preparation and Structural Characterization of CAms

A series of CAms, **1**–**3**, was prepared by setting the molar feed ratios of EPTMAC to CMC to 2:1, 4:1, and 8:1, respectively (Table 1). All the cationization reactions of CMC were conducted in NaOH solutions at 333 K for 24 h. After precipitation with methanol, purification, and then drying under vacuum, CAms **1**–**3** were obtained.

**Table 1:** Initial feed amounts of CMC and EPTMAC used in the synthesis of CAms **1**–**3**, DS_C_, DC_A_, and yields of the obtained CAms.

Sample	Initial Feed Amount		DS_A_	DS_C_	Yields/g (%)
	CMC/g (mmol) ^a^	EPTMAC/g (mmol)			
CAm **1**	12.0 (55.5)	16.8 (111)	0.68	0.41	13.5 (87)
CAm **2**	12.0 (55.5)	33.6 (222)	0.68	0.79	16.9 (91)
CAm **3**	12.0 (55.5)	67.3 (444)	0.68	1.08	18.0 (85)

^a^ molar feed amount of CMC was calculated by the following equation: (Molar amount of CMC) = (amount of CMC/g)/216.4 g·mol^−1^; where 216.4 g·mol^−1^ is the average molecular weight per one anhydroglucose unit of CMC.

**Figure 2:**
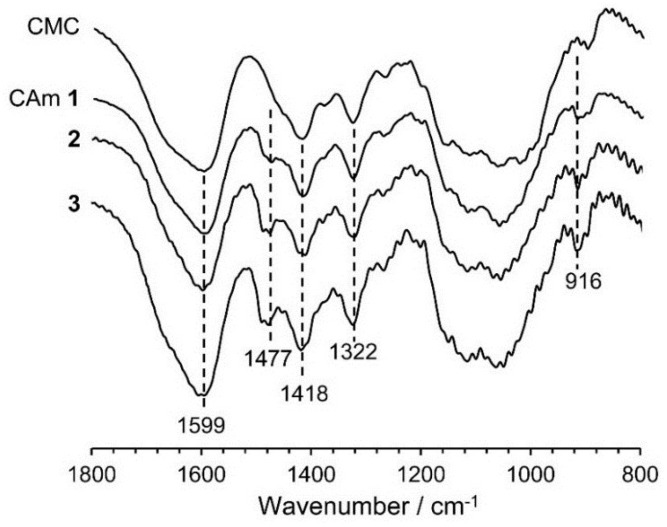
FTIR spectra of CAms **1**‒**3** and CMC.

Figure 2 shows the Fourier transform infrared spectroscopy (FTIR) spectra of CAms **1**–**3** and of the starting material CMC. Common absorption bands are observed at 1599, 1418, and 1322·cm^−1^, which are assigned to the asymmetric stretching of the carboxylate –COO^−^, –CH_2_ scissoring, and the –OH bending vibration, respectively [22]. In addition to these bands, new absorption bands at 1477 and 916·cm^−1^ due to the asymmetric –CH_3_ stretching vibration and the C–N stretching vibration [22], respectively, were detected in the spectra of the CAms, indicating that the EPTMAC was etherified at the hydroxyl groups of CMC, resulting in the substitution of cationic groups on the CMC. In addition, the intensity of the adsorption at 1477·cm^−1^ for the CAm spectra increased with an increase in the feed ratio of EPTMAC to CMC, suggesting that higher concentrations of EPTMAC resulted in increases in DS_C_.

**Figure 3:**
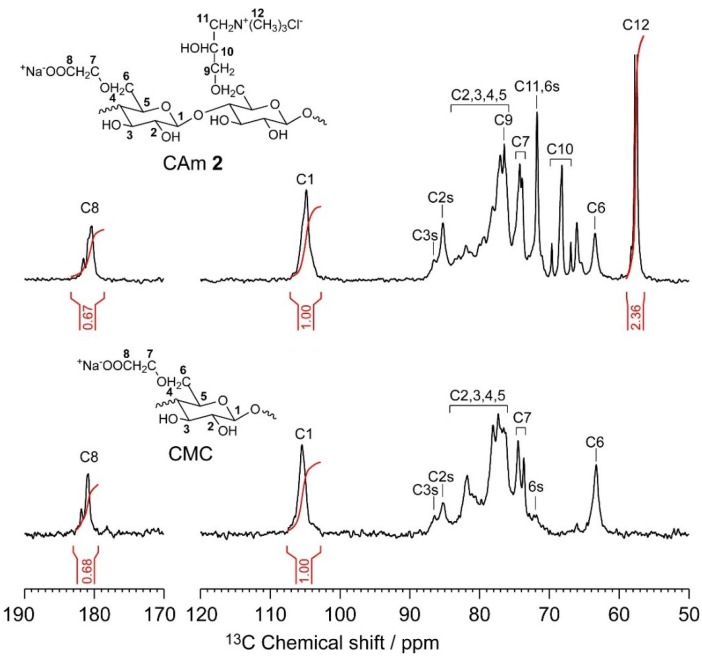
Quantitative ^13^C NMR spectra of CAms **1**‒**3** and CMC in deuterium oxide at 363 K. The C2s, C3s, C6s labels indicate C2, C3, and C6 centers in which hydroxyl groups are substituted by anionic and/or cationic groups, respectively.

The quantitative ^13^C NMR spectra of CMC and CAm **2** in deuterium oxide are shown in Figure 3, which also highlights previous assignments for the ^13^C resonances [22]. As the EPTMAC feed amount used in the preparation of the CAms was increased, the intensity of the 56 ppm peak corresponding to the methyl carbons in the substituted cationic groups was enhanced, while the signal of the carbonyl carbon in the anionic –COO^−^Na^+^ groups at 180 ppm hardly changed. The integrals of the ^13^C signals for the methyl and carbonyl carbons (*I*_C12_ and *I*_C8_, respectively) and for the anomeric carbon at 105 ppm (*I*_C1_) in each spectrum can be used to determine the DS_C_ and DS_A_ according to Equations (1) and (2),

DS_A_ = *I*_C8_/*I*_C1_ (1)

DS_C_ = *I*_C12_/3*I*_C1_ (2)

The DS_C_ and DS_A_ values can be then used to calculate the yield obtained for each CAm through Equation (3),

Yield (%) = Yield (g)/*M*w_AGU_ (g·mol^−1^) × 100/4.6 × 10^−3^ (mol) (3)

where *M*w_AGU_ is the average molecular weight of the anhydroglucose unit (AGU) for each CAm, which can be calculated through Equation (4),

*M*w_AGU_ (g·mol^−1^) = 162 + 151.5 × DS_C_ + 80 × DS_A_ (4)

Table 1 summarizes the DS_C_, DS_A_, and yields of the obtained CAms. The DS_C_ values for CAms **1**–**3** were 0.41, 0.79, and 1.08, respectively, and the DS_A_ of all CAms was 0.68, which is the same as that of CMC. This indicates that the ether-exchange reaction did not occur during the cationization of CMC by EPTMAC. In addition, the yields were in the range of 88%–92%, and are in good agreement with values previously reported [22].

### 2.2. Preparation and Structural Characterization of CAmGs

CAmGs **1**–**3** and CMC hydrogel (CMCG) were prepared from CAms **1**–**3** and CMC, respectively, by a crosslinking reaction with ethyleneglycoldiglycidylether (EGDE) [23,24]. During the reactions, each transparent CAm or CMC solution became increasingly viscous soon after the addition of EGDE, and the morphology of each mixture gradually changed from a solution to a gel after the onset of heating at 60 °C. The crosslinking reaction was allowed to proceed with stirring at 60 °C for 3 h, and the resulting gel was neutralized, purified, and dried under reduced pressure. The dried CAmGs and CMCG obtained were granular white powders.

**Figure 4:**
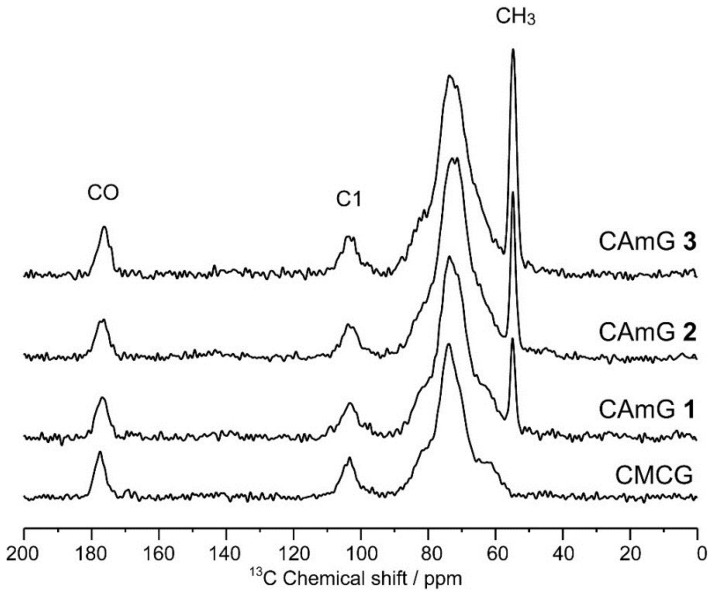
Solid-state DDMAS ^13^C NMR spectra of CAmGs **1**–**3** and CMCG.

Figure 4 shows the solid-state dipolar-decoupled magic angle spinning (DDMAS) ^13^C NMR spectra of CAmGs **1**–**3** and CMCG. The ^13^C resonances of these spectra can be divided broadly into three regions: 185–172, 115–92, and 92–45 ppm. These regions can be assigned, respectively, to the carbonyl carbons, the anomeric C1 of an AGU, and other carbons, which are C2, C3, C4, C5, and C6 of the AGU, the six carbons of the quaternary ammonium groups, the methylene carbon of the carboxymethyl group, and the eight carbons of the EGDE that reacted with each CAm or CMC. Moreover, no unreacted epoxy carbon resonances of EGDE could be observed at 44.9 ppm [25] in these spectra, indicating that the epoxy groups of EGDE at both ends reacted to form the ether crosslink, and that there was no residual graft form of EGDE in CAmGs **1**–**3** or CMCG. To estimate the amounts of EGDE crosslinked with CAmGs **1**–**3** and CMCG, the integration values of the carbonyl carbons (*I*_CO_, 185–172 ppm) and of the other carbon resonances (*I*_others_, 92–45 ppm) of each hydrogel were determined with the integration value of C1 (*I*_C1_, 115–92 ppm) set to one. From the *I*_others_ value for each hydrogel, the average number of the crosslinked EGDEs per one AGU of each CAmG and CMC, which was defined as crosslinking degree (CD), could be calculated through Equation (5):

CD = (*I*_others_ − 6DS_C_ − DS_A_ − 5)/8 (5)

As summarized in Table 2, the ranking of the hydrogels with CD values in decreasing order is as follows: CD_CMCG_ > CD_CAmG **1**_ > CD_CAmG **2**_ > CD_CAmG **3**_. This indicates that a CAm that is highly substituted with cationic groups undergoes less crosslinking with EGDE, since the number of crosslinking points (the free hydroxyl groups) is decreased with an increase in DS_C_. From the CR values, the percent yields of CAmGs **1**–**3** and CMCG could be determined by the Equation (6),

Yield (%) = (yield/g) × 100/(*M*w_AGU_ + (174.2 × CD)) (6)

where the constant, 174.2, is the molecular weight (*M*w) of EGDE, and *M*w_AGU_ is the average molecular weight of each CAm or CMC used for the gel preparation, as defined in the Equation (4). The yields of the CAmGs and CMCG are in range of 82%–89% (Table 2).

**Table 2:** Initial feed amounts of each CAm (or CMC) and EGDE used in the synthesis of CAmGs **1**–**3** and CMCG, and the corresponding yields.

Sample	Initial Feed Amount					CD	Yields/g (%)
	CAm 1/g (mmol) ^a^	CAm 2/g (mmol) ^a^	CAm 3/g (mmol) ^a^	CMC/g (mmol) ^a^	EGDE/g (mmol)		
CAmG **1**	5.0 (18.0)				3.3 (19.2)	0.27	4.8 (82)
CAmG **2**		5.0 (14.9)			3.3 (19.2)	0.24	4.9 (87)
CAmG **3**			5.0 (13.2)		3.3 (19.2)	0.17	4.8 (89)
CMCG				5.0 (23.1)	3.3 (19.2)	0.30	5.1 (82)

^a^ molar feed amounts of CAms and CMC were calculated by the following equation: (Molar amount of CAm or CMC) = (amount of CAm or CMC/g)/*M*w_AGU_ g·mol^−1^; where *M*w_AGU_ of each CAm or CMC was defined in Equation (4).

### 2.3. Water Absorbency of CAmGs

**Figure 5:**
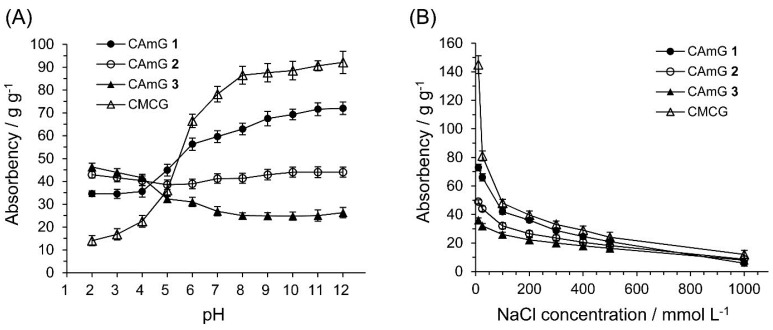
Effects of pH (**A**) and ionic strength (**B**) on the absorbency of CAmGs **1**‒**3** and CMCG at 298 K.

The dried CAmGs **1**–**3** and dried CMCG, consisting of white particles, absorbed water readily, forming transparent hydrogels upon soaking. The absorbencies for these hydrogels reached equilibrium within a few hours (data not shown). Figure 5 shows the absorbency of these hydrogels in solutions with pH values in the range of 2–12 at equilibrium. The absorbency of CMCG increased markedly with increasing buffer pH, due to the presence of carboxyl groups in the structure. In the neutral and alkaline pH regions, because the dominant charged species in CMCG are unprotonated carboxyl groups, CMCG swells due to an intraionic repulsion between the unprotonated carboxyl groups. In the case of acidic pH, on the other hand, the carboxyl groups are protonated, thereby lowering this ionic repulsion, which causes the hydrogel to shrink. In the cases of CAmGs **1**–**3**, the absorbencies strongly depended on the balance of DS_C_ and DS_A_. The cationic groups of the hydrogels are positively charged in the whole pH range of 2–12. However, it is considered that the carboxyl groups of the CAmGs are negatively charged in solutions of pH >4.2, and show electrical neutrality in solutions of pH <4.2, since the pKa of CMC is around 4.2 [25]. Therefore, it is expected that CAmGs act as positively charged hydrogels in acidic solution, and as amphoteric hydrogels in neutral and alkaline solutions. As shown in Figure 5, the absorbencies of all CAmGs are higher than that of CMCG in acidic solutions; a ranking of the CAmGs in order of decreasing absorbency is as follows: CAmG **3** > CAmG **2** > CAmG **1**. This indicates that the swelling of a CAmG in an acidic solution is mainly due to the intracationic repulsion between its cationic groups. In neutral and alkaline solutions, the ranking of the CAmGs by absorbency is changed to following order: CAmG **1** > CAmG **2** > CAmG **3**. This indicates that partial charge neutralization between the anionic and cationic groups occurs in the CAmG hydrogels, because the carboxymethyl groups are negatively charged in these solutions. For example, the anionic property is dominant for CAmG **1** because its DS_A_ is higher than its DS_C_, which results in an increase of absorbency with a pH increase. On the other hand, CAmG **3**, whose DS_A_ and DS_C_ are 0.68 and 1.08, respectively, is positively charged as a whole molecule, and a weak repulsion between the quaternary ammonium cationic groups is the dominant force, which causes the gel to swell with water. CAmG **2**, whose DS_C_ and DS_A_ are 0.79 and 0.68, respectively, showed a behavior between that of CAmGs **1** and **3**.

The effect of ionic strength on the absorbency of CAmGs **1**–**3** and CMCG is also shown in Figure 5. The absorbency of each hydrogel decreased with an increase in the ionic strength of the solution. Because the osmotic pressure caused by the difference in the mobile ion concentration between the inside of a gel and the solution gives rise to the absorbency, a high ionic strength of a solution drastically lowered the absorbency of the CAmGs as well as of CMCG.

### 2.4. Adsorption of Anionic Dyes onto CAmGs

#### 2.4.1. Effects of pH and Cationic Degree of CAmGs

Among the external parameters that can affect the adsorption of dyes onto ionic gels, the pH of the adsorption system is the most important [25,26]. In addition, the charge balance between the cationic and anionic groups of an ionic gel may have a great influence on its dye adsorption [27,28,29]. Therefore, the effects of these parameters on the adsorption of anionic dyes onto CAmGs **1**–**3** and CMCG were assessed using AR9 as the anionic dye.

**Figure 6:**
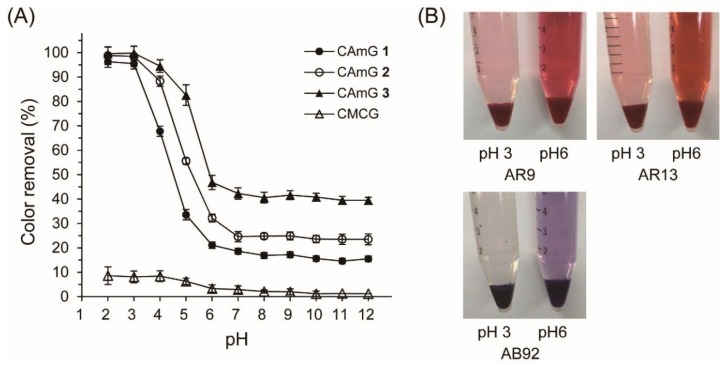
(**A**) Effect of pH on the color removal of AR9 by CAmGs **1**‒**3** and CMCG. The color removal was performed under the following conditions: hydrogel content (dried): 20 mg; AR9 initial concentration: 200 mg/L; 298 K; contact time: 24 h; and volume of AR9 solution: 10 mL; (**B**) Photographs of the adsorption of AR9, AR13, and AB92 onto CAmG **3** at pH 3 and 6.

Figure 6 shows the AR9 color removal by the hydrogels at various pH values ranging between 2 and 12; photographs of the AR9 solutions with CAmG **3** at pH 3 and pH 6 are also shown in this figure. Color removal by CAmGs **1**–**3** reached over 95% at pH values of 2 and 3, an amount that was sharply reduced as the solutions’ pH values increased in value from 4 to 6, and that was almost constant at pH values between 7 and 12. The CAmGs’ ranking in decreasing order of color removal over the whole pH region is as follows: CAmG **3** > CAmG **2** > CAmG **1**. These findings indicate that the adsorption of AR9 was mainly caused by the electrostatic attraction forces between the anionic dye and the cationic groups of the gels, because the carboxymethyl groups of the CAmGs lost negative charge at pH values of 2 and 3 due to protonation, but are charged negatively in neutral and alkaline solutions. This results in a drastic decrease of color removal at higher pH values, due to the electrostatic repulsion between the anionic dye and the anionic carboxymethyl groups. For CMCG, although almost no color removal could be observed in the neutral and alkaline region due to the electrostatic repulsion between the dye and the carboxylate anion of CMCG, only 8% color removal was detected in solutions in the pH range of 2‒4.

#### 2.4.2. Effect of the Adsorbent Dosage

**Figure 7:**
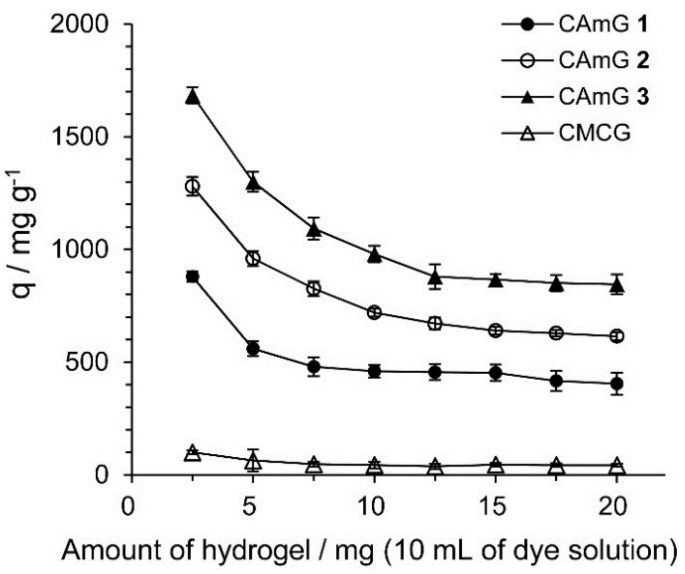
Effect of amount of CAmGs **1**‒**3** and CMCG on the AR9 adsorption. The adsorption experiments were performed under the following conditions: AR9 initial concentration: 200 mg/L; 298 K; contact time: 24 h; and pH 3.

Figure 7 shows the effect of the amount of hydrogels present in solution on their ability to adsorb (*q*) AR9, defined as the amount of adsorbed anionic dyes per gram of hydrogel. The *q* values decreased with an increasing adsorbent dosage, indicating that the smaller the amount of adsorbent present, the more dye of each gel particle came into contact with AR9 (by unit weight of AR9). Similar phenomena were also observed for the adsorption of dyes onto hydrogels prepared from chitosan [30]. The *q* values for CAmGs **1**–**3** strongly depend on the DS_C_ of these hydrogels, and, as expected, hydrogels with higher degrees of cationic substitution adsorbed more anionic dye.

#### 2.4.3. Adsorption Kinetics

**Figure 8:**
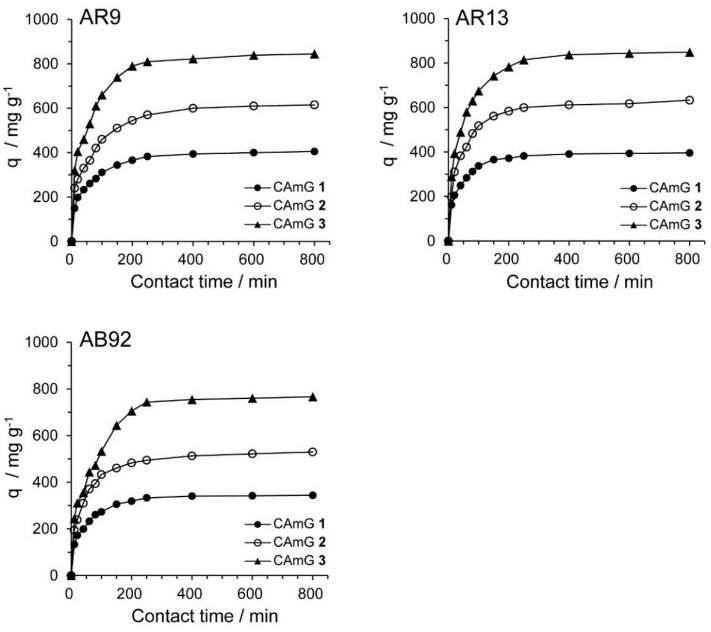
Effect of contact time on the adsorption ability of CAmGs **1**‒**3** toward AR9, AR13, and AB92. The adsorption experiments were performed under the following conditions: hydrogel content (dried): 20 mg; initial concentration of each dye: 200 mg/L; 298 K; pH 3; and volume of each dye solution: 10 mL.

The contact time between the adsorbent and the dye molecules is another important parameter for the adsorption process because it can provide information on the kinetics of the dye for a given initial concentration of adsorbent [20,28]. Therefore, the effect of contact time on the adsorption ability of CAmGs **1**–**3** for AR9, AR13, and AB92 was investigated at pH 3; these data are shown in Figure 8. The adsorption behavior of AR9, AR13, and AB92 onto each CAmG can be divided into three regions: the adsorption rapidly increased in the first 30 min, then gradually increased until equilibrium was reached at a contact time of 250 min, after which it remained constant. The behavior in the first region is due to the rapid diffusion of the anionic dye to the cationic groups on the surface of CAmGs and the subsequent charge neutralization. The second region, with a slower increase in the adsorption of the dyes, is due to the decrease of the effective cationic charge on the CAmGs’ surfaces and the adsorption of dye molecules onto the inside of the gels. After 250 min, the third region begins, where there is almost no further increase in dye adsorption onto the CAmGs, indicating that the equilibrium time for their dye adsorption is about 250 min.

Several kinetic models have been previously used to describe the kinetics of dye adsorption by hydrogels [31], activated carbons [32], and others. Among these models, a pseudo-first order [33] and a pseudo-second order kinetic model [34] have been frequently used to describe dye adsorption onto ionic hydrogels. The pseudo-first order and pseudo-second order kinetic models are expressed by

ln(*q*_e_ − *q*_t_) = ln(*q*_e_) − *k*_1_·*t* (7)

**Figure 9:**
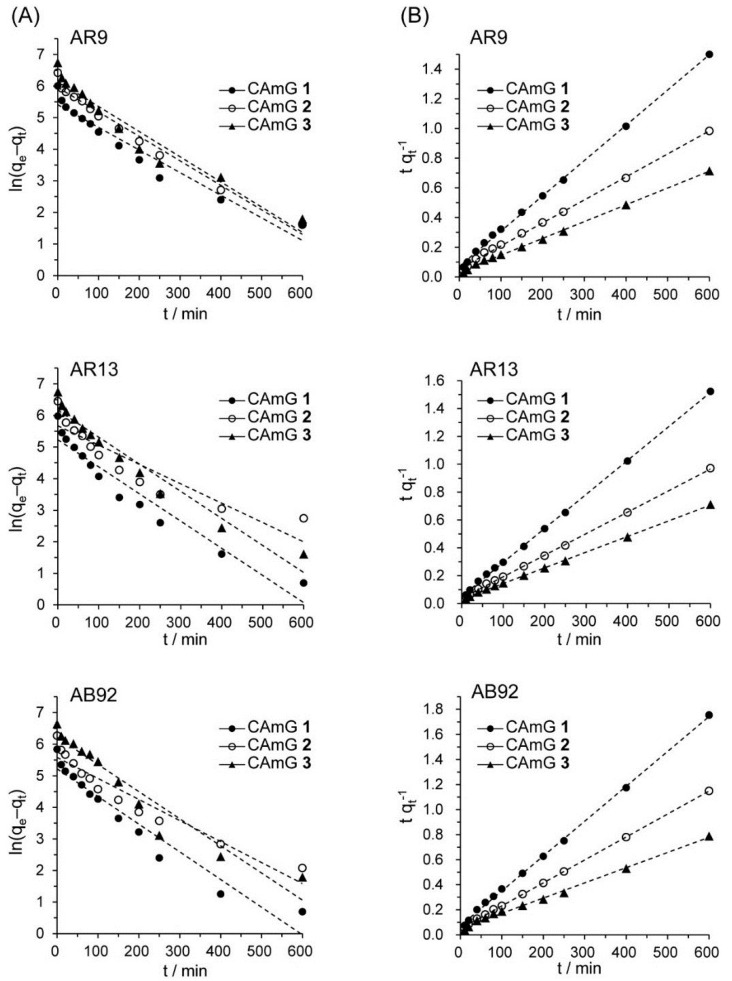
Pseudo-first order (**A**) and pseudo-second order (**B**) kinetic plots for the adsorption of AR9, AR13, and AB92 onto the CAmGs **1**‒**3**.

*t*/*q*_t_ = *k*_2_^−1^·*q*_e_^−2^ + *q*_e_^−1^·*t* (8)

respectively, where *k*_1_ and *k*_2_ are the rate constants for the pseudo-first order and pseudo-second order kinetic models, while *q*_e_ and *q*_t_ are the adsorption abilities of each hydrogel at equilibrium and at contact time *t*, respectively. Based on Figure 8, ln(*q*_e_ − *q*_t_) was plotted *versus* *t* (Figure 9A) and the slopes and intercepts of the plots were used to determine the pseudo-first order rate constant *k*_1_ and the theoretical adsorption ability at equilibrium, *q*_e,cal_. Likewise, to discover if the pseudo-second order model was applicable, the slopes and intercepts of plots of *t*/*q*_t_ *versus* *t* were used to determine the pseudo-second order rate constants *k*_2_ and the corresponding theoretical adsorption abilities at equilibrium *q*_e,cal_ (Figure 9B). The kinetic parameters for the two kinetic models determined from the curve-fitting plots are presented in Table 3 and Table 4. The correlation coefficients (*R*^2^) for the pseudo-second order kinetic model are in the range of 1.000–0.997, considerably higher than those for the pseudo-first order model (*R*^2^ = 0.842–0.980). In Figure 8, the equilibrium time for the adsorption was defined to be 800 min, and each *q*_t_ value at *t* = 800 min was used for *q*_e_. Moreover, it was found that the theoretical *q*_e,cal_ values determined by the pseudo-second order model were more consistent with the experimentally determined *q*_e_ at 800 min, compared with the *q*_e,cal_ determined by the pseudo-first order kinetic model, suggesting that the pseudo-second order adsorption mechanism was predominant for the adsorption of these anionic dyes onto the CAmGs. Similar kinetic behavior has been reported for the electrostatic adsorption of ionic dyes onto polysaccharide-based hydrogels [35,36]. Therefore, it is possible to predict the flocculation behavior of anionic dyes in the presence of CAmGs over the entire range of contact time by using the rate constant values (*k*_2_) determined for the pseudo-second order kinetic model.

**Table 3:** Kinetics parameters for the adsorption of AR9, AR13, and AB92 onto CAmGs **1**‒**3**, determined by fitting to the pseudo-first order kinetic model.

Dye	Adsorbent	*q*_e, *t* = 800 min_^a^/mg·g^−1^	*k*_1_/min^−1^	*q*_e,cal_/mg·g^−1^	*R*^2^
AR9	CAmG **1**	405	0.00716	223	0.943
	CAmG **2**	615	0.00775	386	0.980
	CAmG **3**	845	0.00796	469	0.943
AR13	CAmG **1**	396	0.00859	187	0.935
	CAmG **2**	633	0.00613	291	0.842
	CAmG **3**	849	0.00857	477	0.955
AB92	CAmG **1**	344	0.00877	185	0.941
	CAmG **2**	530	0.00666	266	0.918
	CAmG **3**	767	0.00859	503	0.931

^a^ *q*_t_ value at the contact time of 800 min.

**Table 4:** Kinetics parameters for the adsorption of AR9, AR13, and AB92 onto CAmGs **1**‒**3**, determined by fitting to the pseudo-second order kinetic model.

Dye	Adsorbent	*q*_e, *t* = 800 min_^a^/mg·g^−1^	*k*_2_/g mg^−1^·min^−1^	*q*_e,cal_/mg·g^−1^	*R*^2^
AR9	CAmG **1**	405	8.32 × 10^−5^	411	0.999
	CAmG **2**	615	4.79 × 10^−5^	621	0.998
	CAmG **3**	845	4.15 × 10^−5^	850	0.999
AR13	CAmG **1**	396	1.23 × 10^−5^	398	1.000
	CAmG **2**	633	6.35 × 10^−5^	644	1.000
	CAmG **3**	849	4.36 × 10^−5^	852	0.999
AB92	CAmG **1**	344	1.15 × 10^−5^	346	0.999
	CAmG **2**	530	7.34 × 10^−5^	532	1.000
	CAmG **3**	767	3.28 × 10^−5^	772	0.997

^a^ *q*_t_ value at the contact time of 800 min.

In general, the shape and particle size of the adsorbent affect the diffusion of adsorbate within the adsorbent particles [37]. Therefore, the adsorption kinetics strongly depend on the parameters of the adsorbent. In this study, even if the particle shapes of CAmGs were irregular, adsorption kinetics of anionic dyes onto CAmGs could be fitted by the pseudo-second order kinetic model. This indicated that particles in each CAmG have a different adsorption ability towards anionic dyes and that the adsorption ability of each CAmG was the averaged ability of all particles contained in the hydrogel.

#### 2.4.4. Adsorption Isotherms

**Figure 10:**
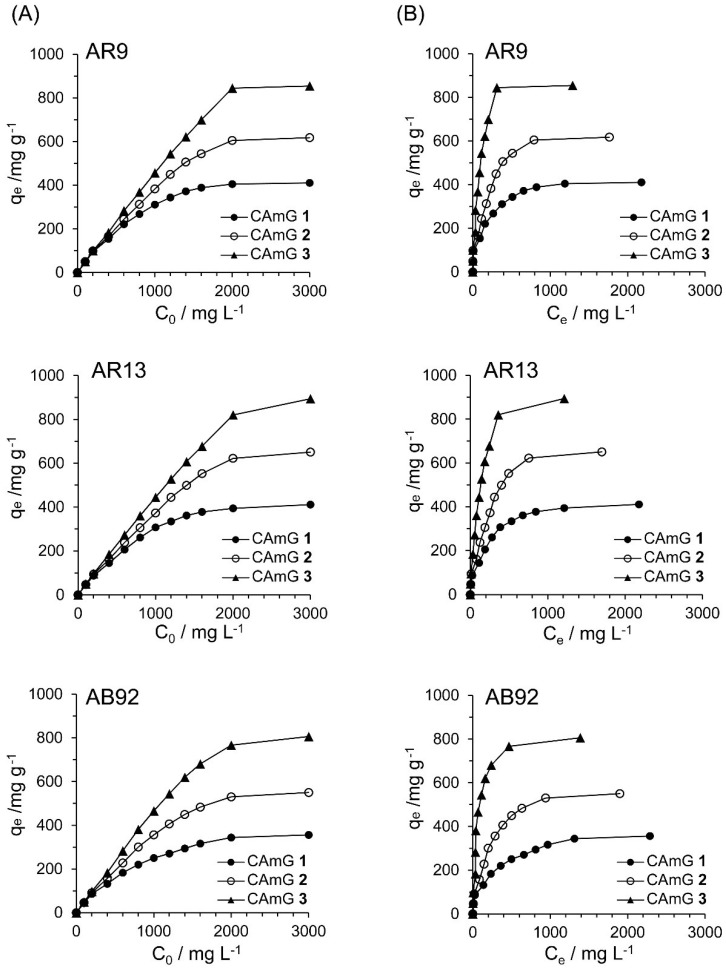
Effects of initial concentration (**A**) and equilibrium concentration (**B**) of AR9, AR13, and AB92 on the *q*_e_ of CAmGs **1**‒**3** toward AR9, AR13, and AB92. The adsorption experiments were performed under the following conditions: hydrogel content (dried): 20 mg; 298 K; pH 3; and volume of each dye solution: 10 mL.

Figure 10A shows the effect of the initial dye concentration (*C*_0_) of dyes AR9, AR13, and AB92 on the flocculation ability at equilibrium (*q*_e_) of each CAmG at pH 3. For all cationic dyes, elevated *q*_e_ values were observed with an increase in *C*_0_, and saturation levels were gradually achieved at initial concentrations of over 2000 mg·L^−1^. In addition, a ranking of the CAmGs according to their saturated *q*_e_ values for all dyes, in increasing order, is CAmG **1** < CAmG **2** < CAmG **3**, indicating that the anionic dyes were adsorbed via the charge neutralization mechanism.

Adsorption is commonly described through the use of isotherms, meaning the amount of adsorbate on the adsorbent as a function of pressure, in the case of a gas, or concentration, in the case of a liquid, at a constant temperature. The adsorption isotherm is important for describing the interaction of the adsorbates with the adsorbents, and for optimizing the use of adsorbents. Among a number of isotherm models, the Langmuir and Freundlich ones [35,36,38,39], which are frequently used to model the adsorption of dye onto polymer surfaces, were applied here to the adsorption of the anionic dyes by CAmGs **1**‒**3**.

The basic assumption of the Langmuir isotherm model is that adsorption occurs at specific homogeneous sites on the adsorbent. It is further assumed that once an adsorbate occupies a site, no further adsorption can take place at that site [40]. Therefore, the linear form of the Langmuir equation can be expressed by

*q*_e_^−1^ = *Q*_0_^−1^ + (b·*Q*_0_·*C*_e_)^−1^ (9)

where *q*_e_ is amount of adsorbate on the adsorbent at equilibrium (mg·g^−1^), *Q*_0_ is the adsorbate’s maximum monolayer coverage capacity (mg·g^−1^), *b* is the Langmuir isotherm constant (L·mg^−1^), and *C*_e_ is the equilibrium concentration of adsorbate (mg·L^−1^) in solution. Moreover, a dimensionless constant, commonly known as the separation factor (*R*_L_) defined by Weber and Chakravorti [39], can be represented as

*R*_L_ = (1 + *b*·*C*_0_)^−1^ (10)

Based on its *R*_L_ value, an adsorption process can be categorized as unfavorable (*R*_L_ > 1), linear (*R*_L_ = 1), favorable (0 < *R*_L_ < 1), or irreversible (*R*_L_ = 0). On the other hand, the linearized form of the Freundlich isotherm equation is given by [39],

ln(*q*_e_) = ln*K*_F_ + *n*^−1^·ln*C*_e_ (11)

where *K*_F_ is the Freundlich isotherm constant, which relates to the adsorption capacity, and the dimensionless constant *n*^−1^ is an empirical constant, which gives valuable information about the isotherm’s shape. Based on the *n*^−1^ values, the adsorption process may be classified as irreversible (*n*^−1^ = 0), favorable (0 < *n*^−1^ < 1), or unfavorable (*n*^−1^ > 1).

**Figure 11:**
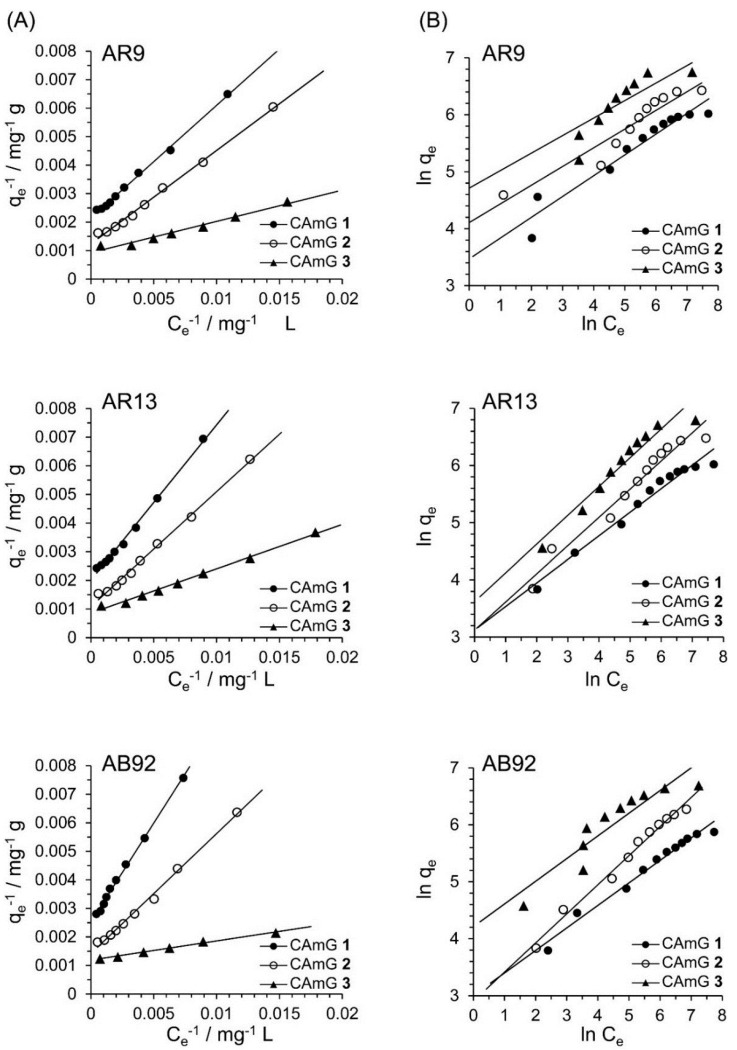
Langmuir isotherm (**A**) and Freundlich isotherm (**B**) for the encapsulation of AR9, AR13, and AB92 adsorption in the CAmGs at pH 3 and 298 K.

Based on Figure 10A, the effects of the equilibrium concentrations of the anionic dyes on the adsorption abilities of CAmGs **1**‒**3** are shown in Figure 10B, and linear plots corresponding to the Langmuir and Freundlich isotherm models for the adsorption of each anionic dye on the CAmGs are presented in Figure 11. The linearized isotherm coefficients for these models, determined from the plots, are summarized in Table 5 and Table 6. The values of the dimensionless constant *R*_L_ obtained from the Langmuir model fall in range of 0.115–0.255, indicating the favorable adsorption of all of the anionic dyes onto each CAmG. Additionally, for the Freundlich model, the values of the empirical constant *n*^−1^ lie in the range of 0.655–0.908, also indicating the favorable adsorption of the dyes onto the CAmGs. However, the correlation coefficients *R*^2^ for the Langmuir model are in range of 0.996–0.999, while those for the Freundlich model are lower, in the range of 0.942–0.991. In addition, as shown in Table 5, the maximum monolayer coverage capacities (*Q*_0_) of the CAmGs for the anionic dyes were in good agreement with the equilibrium adsorption abilities of the CAmGs. These data imply that the adsorption of the anionic dyes onto the CAmGs follows a Langmuir-type adsorption mechanism. The values of the Langmuir constant b, which represents the constant of dissociation of adsorbed dye molecules to free anionic dyes, are on the order of 10^−2^–10^−3^, indicating that the flocculi formed by the anionic dyes and the CAmGs are very stable in solution.

**Table 5:** Langmuir isotherm coefficients for the adsorptions of AR9, AR13, and AB92 onto CAmGs.

Dye	Adsorbent	*Q*_0_/mg·g^−1^	*b*/mg^−1^·L	*R*_L_	*R*^2^
AR9	CAmG **1**	419	0.00604	0.0523	0.997
	CAmG **2**	644	0.00474	0.0658	0.996
	CAmG **3**	866	0.01051	0.0307	0.976
AR13	CAmG **1**	410	0.00450	0.0690	0.996
	CAmG **2**	651	0.00383	0.0801	0.996
	CAmG **3**	911	0.00710	0.0448	0.994
AB92	CAmG **1**	371	0.00389	0.0789	0.997
	CAmG **2**	561	0.00424	0.0729	0.996
	CAmG **3**	816	0.01831	0.0179	0.994

**Table 6:** Freundlich isotherm coefficients for the adsorptions of AR9, AR13, and AB92 onto CAmGs.

Dye	Adsorbent	*K*_F_/mg·L^−1^	*n*^−1^	*R*^2^
AR9	CAmG **1**	32.1	0.365	0.931
	CAmG **2**	60.8	0.328	0.956
	CAmG **3**	111.4	0.307	0.866
AR13	CAmG **1**	22.7	0.412	0.968
	CAmG **2**	22.6	0.493	0.961
	CAmG **3**	37.2	0.504	0.934
AB92	CAmG **1**	20.2	0.395	0.977
	CAmG **2**	18.4	0.510	0.986
	CAmG **3**	67.5	0.398	0.844

#### 2.4.5. Adsorption Mechanism

As discussed in the preceding section, the adsorption of anionic dyes onto CAmGs **1**‒**3** follows the Langmuir adsorption isotherm model. This adsorption model is constructed based on the following assumptions [39]:

- The entire surface of the adsorbent is uniform, and all the adsorption sites are equivalent.
- There is no interaction between the adsorbed molecules.
- All adsorbate molecules absorb onto the adsorbent by the same mechanism.
- The adsorbate molecules adsorb only onto the surface of the adsorbent, not onto previously adsorbed molecules.

Therefore, the adsorption of dye molecules onto the CAmGs is a homogeneous mechanism based on the charge neutralization between the dye molecules and the cationic surface of the CAmGs. This suggests that the hydrophobic interaction between the dye molecules, for example, π–π stacking interactions between aromatic rings of the anionic dyes [40], is so small as to be negligible compared with the electrostatic interaction between the anionic dyes and the cationic groups of the CAmGs. If such an interaction played an important role in the adsorption of anionic dye, apart from the electrostatic interaction, the adsorption isotherm would fit or be close to the Freundlich adsorption isotherm model, because the Freundlich isotherm is generally used to describe heterogeneous adsorption systems.

Except for the adsorption phenomenon of anionic dyes at cationic sites of the CAmGs, the adsorption mechanisms resulting from these systems are rather complex: the adsorption mechanisms are related to the swelling of CAmGs, the diffusion of dyes from the dye medium toward the hydrogel mass. Lamberti *et al.* and Cacavavo *et al.* reported that the swelling/diffusion process can be mathematically described using a physical model proposed by their groups [41,42,43]. Therefore, theoretical approaches for the phenomena including gel swelling as well as dye diffusion are required to precisely reveal the adsorption mechanism.

## 3. Conclusions

In this study, a series of CAms was prepared from CMC by its etherification with EPTMAC under aqueous alkaline solution, and the CAms were converted to CAmGs by use of EGDE as a crosslinking agent. The CAmGs exhibited swelling behavior in buffer solutions with a wide range of pH values. The adsorption abilities of the CAmGs towards three cationic dyes were investigated, from which we draw the following three findings:

1. The adsorption ability of a CAmG strongly depends on its DS_C_ and on the pH of the adsorption medium: the maximum adsorption of anionic dyes occurred using CAmGs with higher DS_C_ values, and under lower pH (less than 3) conditions.
2. The adsorption of AR9, AR13, and AB13 onto the CAmGs occurs via a pseudo-second order kinetic mechanism.
3. The adsorption isotherms of the three anionic dyes by the CAmGs could be well fitted by the Langmuir adsorption isotherm model rather than the Freundlich adsorption isotherm model, indicating that the adsorption of the anionic dyes onto the CAmGs predominantly occurred via the charge neutralization mechanism.

Based on these findings, it can be concluded that the CAmGs are highly capable of the adsorption of anionic dyes and may be suitable remediation adsorbents that can readily remove anionic dyes in solution. In addition, the CAmGs could be obtained at relatively low cost because cellulosic biomass could be used as a raw material. Therefore, CAmGs are expected to be environmentally friendly adsorbents for wastewater treatment, and important candidates to potentially replace synthetic polymer adsorbents, activated carbons, and other currently used adsorbent materials.

## 4. Experimental Section

### 4.1. Materials

CMC with DS_A_ = 0.68 and an average molecular weight (*M*w) of 2.6 × 10^5^ was obtained from Daiichi Kogyo Seiyaku Co., Kyoto, Japan. 2,3-Epoxypropyl trimethyl ammonium chloride (EPTMAC) was purchased from Sigma-Aldrich (USA). Ethyleneglycoldiglycidylether (EGDE), AR9, AR13, and AB92 were all purchased from Tokyo Chemical Industry Co. Ltd., Tokyo, Japan. The molecular weights of AR9, AR13, and AB92 were 400.4, 502.4, and 695.6 g·mol^−1^, respectively. Other chemicals were of a chemically pure grade, and all solutions were prepared with pure water.

### 4.2. Preparation of CAms

A series of three CAms, **1**–**3**, were prepared from CMC with EPTMAC according to the method previously reported [44]. Briefly, CMC (12 g) was dissolved in 100 mL of a 1.5 mmol·L^−1^ NaOH solution at 4 °C, and then EPTMAC (16.8 g, 111 mmol) was added dropwise over a period of 20 min. The reaction mixture was heated at 333 K for 24 h. After cooling to room temperature, the reaction mixture was poured into 300 mL of methanol. The obtained precipitates were washed with a 1:1 mixture of methanol/distilled water to reach a neutral pH, dried under vacuum, and then milled and screened through a 40 mesh sieve to obtain CAm **1**. By a similar method, CAms **2** and **3** were prepared by changing the feed amount of EPTMAC; the amounts of EPTMAC for the preparation of CAms **2** and **3** are summarized in Table 1. Obtained CAms **1**–**3** were stored in a desiccator under vacuum.

### 4.3. Preparation of CAmGs

A series of CAmGs, **1**–**3**, were prepared from CAms **1**–**3**, respectively, with EGDE in an organic synthesizer, Process Station PPS-2511, with a Teflon stirring impeller (Tokyo Rikakikai Co., Ltd., Tokyo, Japan). These gels were typically prepared as follows: CAm **1** (5 g) was completely dissolved in a 1.5 M NaOH solution (25 mL) at 277 K using the Teflon impeller at 300 rpm. EGDE (3.3 g, 19.2 mmol) was added to the CAm solution, and the crosslinking reaction was carried out at 333 K for 3 h with continuous stirring. The mixture was added to 100 mL of methanol to precipitate the gel. The gel was washed three times in a 1:1 mixture of methanol/distilled water, dialyzed against water using dialysis tubing (*M*w 1.2–1.3 × 10^4^ cut off, Thermo Fisher Scientific Inc., Waltham, USA) for three days until neutral, and then precipitated with methanol. The precipitate was dried under reduced pressure at 323 K. The resultant solid particles were cut and screened through a 40 mesh filter using a PLC-2M plastic cutting mill (Osaka Chemical Co., Osaka, Japan) to obtain a white granular product of CAmG **1**. CAmGs **2** and **3** were obtained using a similar method. In addition, CMCG was prepared from CMC by a similar method for use as a reference sample. The initial feed amounts of the CAms, CMC, and EGDE used for the preparation of the hydrogels are listed in Table 2. All the obtained gels were stored in a vacuum desiccator until further use.

### 4.4. FTIR Spectroscopy

Each sample was diluted in powdered potassium bromide and compressed into transparent disks. The FTIR spectra of the resulting samples were recorded at 298 K on a PerkinElmer Spectrum II FTIR spectrometer at a resolution of 1 cm^−1^. The samples were scanned from 4000 to 500 cm^−1^ using an average of 16 scans.

### 4.5. NMR Spectroscopy

All the NMR experiments were performed on a Bruker AVIII spectrometer (Bruker BioSpin GmbH, Rheinstetten, Germany) at a ^1^H frequency of 500.13 MHz and a ^13^C frequency of 125.13 MHz. Quantitative solution-state ^13^C NMR spectra were obtained in deuterium oxide at 363 K using a two-channel 5 mm broadband observe probe with a z-gradient coil, by an inverse-gated ^1^H decoupling method [45]. The ^13^C excitation pulse (flip angle of 30°), repetition time, and number of scans were set to 3.3 μs, 45 s, and 2048, respectively. The ^13^H NMR chemical shifts were calibrated by referencing the methyl resonance of the internal standard 4,4-dimethyl-4-silapentane-1-sulfonic acid to 0 ppm. Solid-state ^13^C NMR spectra were obtained at 298 K with a 4 mm dual-tuned MAS probe at 8–10 kHz. Dipolar-decoupled magic angle spinning (DDMAS) ^13^C NMR spectra were recorded by setting the ^13^C excitation pulse length (flip angle of 30°), data acquisition time, and repetition time of 1.35 μs, 15 ms, and 30 s, respectively. SPINAL 64 ^1^H decoupling sequences [46] with a radio-field strength of 75 kHz were used during acquisition. The ^13^C NMR chemical shifts were calibrated by setting the carbonyl carbon resonance of the external standard d-glycine to 176.03 ppm.

### 4.6. Water Absorbency

Dried CAmG or CMCG (*ca*. 100 mg) was precisely weighed and then placed into a 50 mL polypropylene centrifuge tube. The desired solution was poured into the tube, and the tube was set in a shaker at 298 K and shaken at 120 rpm. After 24 h, the tube was centrifuged at 4000× *g* for 10 min and the supernatant was completely drained. The absorbency of the gel could be determined using the following equation,

Absorbency = (*W*_s_ − *W*_d_)/*W*_d_ (12)

where *W*_s_ and *W*_d_ are the weights of the swollen and the dried hydrogel, respectively. The effect of a solution’s pH on the hydrogels’ absorbencies was investigated in 20 mmol·L^−1^ HCl-KCl (pH 2), 20 mmol·L^−1^ citric acid-sodium citrate (pH 3‒6), 20 mmol·L^−1^ NaH_2_PO_4_-Na_2_HPO_4_ (pH 7‒8), 20 mmol·L^−1^ Tris-HCl (pH 9), 20 mmol·L^−1^ NaHCO_3_-NaOH (pH 10–11), and 20 mmol·L^−1^ NaCl-NaOH (pH 12). The effect of ionic strength on the absorbencies was investigated in 10–1000 mmol·L^−1^ solutions.

### 4.7. Dye Adsorption

Three kinds of anionic dyes, whose structures are shown in Figure 1, were used for the adsorption experiments. A buffer solution (5 mL) containing a dye with a concentration in the range of 100–3000 mg·L^−1^ was poured into a 15 mL polypropylene centrifuge tube, and a CAmG or CMCG was added to the dye solution. The mixture was immediately placed in a shaker at 120 rpm and 298 K for a predefined time, after which the suspension was left to settle for 5 min. The dye concentration of the filtrate was determined by an Epoch 96-well micro-volume spectrophotometer (BioTek Instruments, Inc., Winooski, VT, USA) with a 96-well microplate, using calibration curves prepared earlier. The percent removal of each dye and the flocculation ability (*q*_t_) of each CAm at contact time *t* were calculated using Equations (13) and (14), respectively:

(Dye removal) % = (*C*_0_ − *C*_t_)/*C*_0_ × 100 (13)

*q*_t_ = (*C*_0_ − *C*_t_) × *V*/*m* (14)

where *C*_0_ is the initial concentration of the dye solution, *C*_t_ is the concentration of the dye solution at contact time *t*, *V* is the volume of the dye solution, and m is the mass of each CAm. Buffer solutions used for the adsorption media were 20 mmol·L^−1^ citric acid-sodium citrate (pH 3‒6), 20 mmol·L^−1^ NaH_2_PO_4_-Na_2_HPO_4_ (pH 7‒8), 20 mmol·L^−1^ Tris-HCl (pH 9), and 20 mmol·L^−1^ NaHCO_3_-NaOH (pH 10).

## References

[1] Sethuraman V.V., Raymahashay B.C. (1975). Color removal by clays. Kinetic study of adsorption of cationic and anionic dyes. Environ. Sci. Technol..

[2] Yagub M.T., Sen T.K., Afroze S., Ang H.M. (2014). Dye and its removal from aqueous solution by adsorption: A review. Adv. Colloid Interface Sci..

[3] Sen T.K., Afroze S., Ang H.M. (2011). Equilibrium, kinetics and mechanism of removal of methylene blue from aqueous solution by adsorption onto pine cone biomass of Pinus radiate. Water Air Soil Pollut..

[4] Yagub M.T., Sen T.K., Ang H.M. (2012). Equilibrium, kinetics, and thermodynamics of methylene blue adsorption by pine tree leaves. Water Air Soil Pollut..

[5] Fu Y., Viraraghavan T. (2001). Fungal decolorization of dye waste waters: A review. Bioresour. Technol..

[6] Lazar T. (2005). Color chemistry: Synthesis, properties, and applications of organic dyes and pigments, 3rd revised edition. Color Res. Appl..

[7] Kadirvelu K., Kavipriya M., Karthika C., Radhika M., Vennilamani N., Pattabhi S. (2003). Utilization of various agricultural wastes for activated carbon preparation and application for the removal of dyes and metal ions from aqueous solutions. Bioresour. Technol..

[8] Azbar N., Yonar T., Kestioglu K. (2004). Comparison of various advanced oxidation processes and chemical treatment methods for COD and color removal from a polyester and acetate fiber dyeing effluent. Chemosphere.

[9] Mohan S.V., Sailaja P., Srimurali M., Karthikeyan J. (1998). Color removal of monoazo acid dye from aqueous solution by adsorption and chemical coagulation. Environ. Eng. Policy.

[10] Verma A.K., Dash R.R., Bhunia P. (2012). A review on chemical coagulation/flocculation technologies for removal of colour from textile wastewaters. J. Environ. Manag..

[11] Lau Y.Y., Wong Y.S., Teng T.T., Morad N., Rafatullah M., Ong S.A. (2014). Coagulation-flocculation of azo dye Acid Orange 7 with green refined laterite soil. Chem. Eng. J..

[12] Galindo C., Jacques P., Kalt A. (2001). Photochemical and photocatalytic degradation of an indigoid dye: A case study of acid blue 74 (AB74). J. Photochem. Photobiol. A Chem..

[13] Malik P.K. (2004). Dye removal from wastewater using activated carbon developed from sawdust: Adsorption equilibrium and kinetics. J. Hazard. Mat..

[14] Rocher V., Siaugue J.M., Cabuil V., Bee A. (2008). Removal of organic dyes by magnetic alginate beads. Water Res..

[15] Mahdavinia G.R., Bazmizeynabad F., Seyyedi B. (2015). Kappa-Carrageenan beads as new adsorbent to remove crystal violet dye from water: Adsorption kinetics and isotherm. Desalination Water Treat..

[16] Pavan F.A., Mazzocato A.C., Gushikem Y. (2008). Removal of methylene blue dye from aqueous solutions by adsorption using yellow passion fruit peel as adsorbent. Bioresour. Technol..

[17] Crini G., Badot P.-M. (2008). Application of chitosan, a natural aminopolysaccharide, for dye removal from aqueous solutions by adsorption processes using batch studies: A review of recent literature. Prog. Polym. Sci..

[18] Annadurai G., Juang R.S., Lee D.J. (2002). Use of cellulose-based wastes for adsorption of dyes from aqueous solutions. J. Hazard. Mat..

[19] Gupta V.K., Suhas (2009). Application of low-cost adsorbents for dye removal—A review. J. Environ. Manag..

[20] Yang Z., Shang Y., Lu Y., Chen Y., Huang X., Chen A., Jiang Y., Gu W., Qian X., Yang H. (2011). Flocculation properties of biodegradable amphoteric chitosan-based flocculants. Chem. Eng. J..

[21] Kono H., Fujita S., Oeda I. (2013). Comparative study of homogeneous solvents for the esterification crosslinking of cellulose with 1,2,3,4-butanetetracarboxylic dianhydride and water absorbency of the reaction products. J. Appl. Polym. Sci..

[22] Kono H., Kusumoto R. (2014). Preparation, structural characterization, and flocculation ability of amphoteric cellulose. React. Funct. Polym..

[23] Kono H., Otaka F., Ozaki M. (2014). Preparation and characterization of guar gum hydrogels as carrier materials for controlled protein drug delivery. Carbohydr. Polym..

[24] Kono H., Hara H., Hashimoto H., Shimizu Y. (2015). Nonionic gelation agents prepared from hydroxypropyl guar gum. Carbohydr. Polym..

[25] Kono H. (2014). Characterization and properties of carboxymethyl cellulose hydrogels crosslinked by polyethylene glycol. Carbohydr. Polym..

[26] Hadi A.G. (2013). Dye removal from colored textile wastewater using synthesized chitosan. Int. J. Sci. Technol..

[27] Yan L., Tao H., Bangal P.R. (2009). Synthesis and flocculation behavior of cationic cellulose prepared in a NaOH/urea aqueous solution. Clean Soil Air Water.

[28] Singh R.P., Pal S., Rana V.K., Ghorai S. (2013). Amphoteric amylopectin: A novel polymeric flocculant. Carbohydr. Polym..

[29] Lin Q., Qian S., Li C., Pan H., Wu Z., Liu G. (2012). Synthesis, flocculation and adsorption performance of amphoteric starch. Carbohydr. Polym..

[30] Dotto G.L., Pinto L.A.A. (2011). Adsorption of food dyes onto chitosan: Optimization process and kinetic. Carbohydr. Polym..

[31] Koner S., Saha B.K., Kumar R., Adak A. (2011). Adsorption kinetics and mechanism of methyl orange dye on modified silica gel factory waste. Int. J. Sci. Technol..

[32] Hameed B.H., Din A.T.M., Ahmad A.L. (2007). Adsorption of methylene blue onto bamboo-based activated carbon: Kinetics and equilibrium studies. J. Hazard. Mat..

[33] Ho Y.S., McKay G. (1998). Sorption of dye from aqueous solution by peat. Chem. Eng. J..

[34] Ho Y.S., McKay G. (1999). Pseudo-second order model for sorption processes. Process Biochem..

[35] Dalaran M., Emik S., Güçlü G., İyim T.B., Özgümüş S. (2011). Study on a novel polyampholyte nanocomposite superabsorbent hydrogels: Synthesis, characterization and investigation of removal of indigo carmine from aqueous solution. Desalination.

[36] Kono H., Nakamura T., Hashimoto H., Shimizu Y. (2015). Characterization, molecular dynamics, and encapsulation ability of β-cyclodextrin polymers crosslinked by polyethylene glycol. Carbohydr. Polym..

[37] Omidian H., Rocca J.G., Park K. (2005). Advances in superporous hydrogel. J. Control. Release.

[38] Crini G. (2008). Kinetic and equilibrium studies on the removal of cationic dyes from aqueous solution by adsorption onto a cyclodextrin polymer. Dyes Pigment..

[39] Weber T.W., Chakravorti R.K. (1974). Pore and solid diffusion models for fixed-bed adsorbers. AIChE J..

[40] Chipot C., Jaffe R., Maigret B., Pearlman D.A., Kollman P.A. (1996). Benzene dimer: A good model for π−π interactions in proteins? A comparison between the benzene and the toluene dimers in the gas phase and in an aqueous solution. J. Am. Chem. Soc..

[41] Caccavo D., Cascone S., Lamberti G., Barba A.A. (2015). Modeling the drug release from hydrogel-based matrices. Mol. Pharm..

[42] Caccavo D., Cascone S., Lamberti G., Barba A.A. (2015). Controlled drug release from hydrogel-based matrices: Experiments and modeling. Int. J. Pharm..

[43] Lamberti G., Galdi I., Barba A.A. (2011). Controlled release from hydrogel-based solid matrices. A model accounting for water up-take, swelling and erosion. Int. J. Pharm..

[44] Kono H., Kusumoto R. (2015). Removal of anionic dyes in aqueous solution by flocculation with cellulose ampholytes. J. Water Process Eng..

[45] Kono H., Hashimoto H., Shimizu Y. (2015). NMR characterization of cellulose acetate: Chemical shift assignments, substituent effects, and chemical shift additivity. Carbohydr. Polym..

[46] Fung B.M., Khitrin A.K., Ermolaev K. (2000). An improved broadband decoupling sequence for liquid crystals and solids. J. Magn. Reson..

